# A unified concatenation model for plasma physics: Integrability and soliton solutions

**DOI:** 10.1016/j.mex.2025.103641

**Published:** 2025-09-24

**Authors:** Elsayed M.E. Zayed, Mona El–Shater, Ahmed H. Arnous, Anjan Biswas

**Affiliations:** a Department of Mathematics, Faculty of Science, Zagazig University, Zagazig 44519, Egypt; b Department of Mathematical Sciences, Saveetha School of Engineering, SIMATS, Chennai 602105, Tamilnadu, India; c Research Center of Applied Mathematics, Khazar University, Baku, AZ 1096, Azerbaijan; d Department of Mathematics & Physics, Grambling State University, Grambling, LA 71245–2715, USA; e Department of Physics and Electronics, Khazar University, Baku, AZ–1096, Azerbaijan; f Department of Applied Sciences, Cross–Border Faculty of Humanities, Economics and Engineering, Dunarea de Jos University of Galati, 111 Domneasca Street, Galati 800201, Romania; g Department of Mathematics and Applied Mathematics, Sefako Makgatho Health Sciences University, Medunsa 0204, South Africa

**Keywords:** Solitons, Modified sub–ODE, Jacobi, Weierstrass

## Abstract

In numerous fields of mathematical physics, including nuclear physics, fluid dynamics, quantum optics, and plasma physics, the idea of nonlinear evolution equation has left an enduring impression. The concept of concatenation model has recently gained its popularity after its first appearance during 2014. Such a model was proposed in nonlinear optics and exists in two forms: the concatenation model and the dispersive concatenation model, both of which depend on the fundamental components concatenated for their formulation. Likewise, the current paper proposes a concatenation model from plasma physics whose fundamental components are the Kaup–Newell equation, Chen–Lee–Liu equation and the Gerdjikov–Ivanov equation. These encompass various concepts such as Langmuir waves, Alfvén waves, and cold plasmas, which are commonly studied in plasma physics. The special cases of this newly structured concatenation model are apparent as discussed in detail in the subsequent section.

Next, the model’s integrability is examined. For this recently developed model, the soliton solutions are obtained using two integration techniques. The methods are the enhanced direct algebraic method and the modified sub-ODE (ordinary differential equation) approach. These two approaches use the intermediary Jacobi’s and Weierstrass’ elliptic functions, respectively, to obtain the soliton solutions. Solitons can be used to identify special cases of these functions. We utilize the parameter constraints that naturally arise from the two integration approaches. Following an introduction to the model, these are covered in detail in the remaining text.

The concatenated DNLS model formulated here offers a single parameterized framework in which KN, CLL and GI arise as embedded limits; the tunable derivative couplings $\left(c_{1},c_{2},c_{3}\right)$ and higher-order amplitudinal terms $\left(b_{1},b_{2}\right)$ enable controlled passage between convective self-steepening, mixed derivative nonlinearities and quintic saturation. This structure is novel in providing a unified description that consolidates previously separate DNLS-type models into a single tractable form, thereby enabling systematic exploration of plasma nonlinearities across distinct physical regimes.

• The paper proposes a novel concatenation model in plasma physics constructed from three fundamental equations—the Kaup–Newell, Chen–Lee–Liu, and Gerdjikov–Ivanov equations—representing key plasma wave phenomena such as Langmuir and Alfvén waves.
• Soliton solutions of the model are analytically derived using two powerful integration techniques: the enhanced direct algebraic method (involving Jacobi elliptic functions) and the modified sub-ODE method (utilizing Weierstrass elliptic functions).
• The study identifies integrability conditions and parameter constraints from both solution approaches, offering insight into special cases of the model and contributing to the theoretical understanding of nonlinear plasma wave dynamics.

## Graphical abstract

**Figure:**
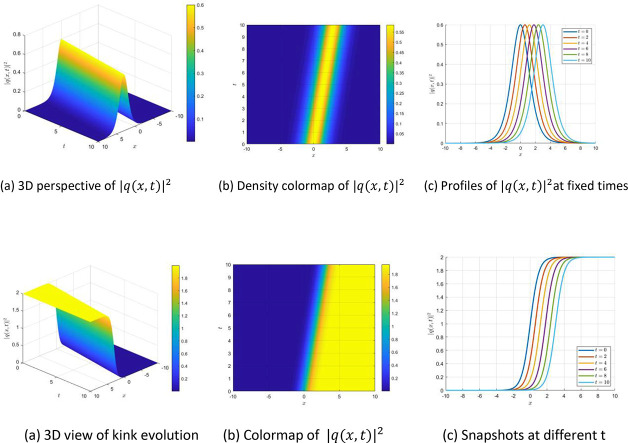

**Specifications table**

**Table:** 

**Subject area**	Mathematics and Statistics
**More specific subject area**	Mathematical Physics
**Name of your method**	The modified sub-ODE and the enhanced direct algebraic method.
**Name and reference of original method**	1 E. M. E. Zayed, M. E. M. Alngar, "Application of newly proposed sub-ODE method to locate chirped optical solitons to Triki–Biswas equation", Optik, V 207, 164360 (2020). 2 A. H. Arnous, M. S. Hashemi, K. S. Nisar, M. Shakeel, J. Ahmad, I. Ahmad, R. Jan, A. Ali, M. Kapoor, N. A. Shah "Investigating Solitary Wave Solutions with Enhanced Algebraic Method for New Extended Sakovich Equations in Fluid Dynamics" Results in Physics V 57, 107369, (2024).
**Resource availability**	Mathematica & Maple

## Background

Nonlinear evolution equations (NLEEs) play a fundamental role in modeling a wide spectrum of physical systems, including optics, plasmas, fluid dynamics, and material sciences. Over the last few decades, they have provided a universal language to describe nonlinear pulse propagation, solitary waves, and rogue-wave phenomena. For example, investigations on shallow water dynamics and conformable models have revealed how analytical soliton solutions capture both solitary and dispersive behaviors in nonlinear waveguides and cold plasma analogues [1]. Likewise, general explorations of analytical approaches to NLEEs further highlight their relevance across multiple domains [2].

A particularly important class of NLEEs are the derivative nonlinear Schrödinger-type equations, which incorporate higher-order nonlinear effects through derivative terms. Among these, the Kaup–Newell (KN), Chen–Lee–Liu (CLL), and Gerdjikov–Ivanov (GI) equations have emerged as prototypical models describing nonlinear pulse dynamics in dispersive and plasma media. The CLL equation, in particular, has been widely used in the context of nonlinear optics and fiber systems, where soliton solutions help explain monomode fiber propagation [3]. Extensions of these models have also appeared in ferromagnetic systems and other complex media, emphasizing the broad applicability of derivative NLS-type equations [4]. Such developments establish these equations as natural candidates for concatenation models that unify their mathematical structures under a common framework.

In parallel, recent years have witnessed extensive study of higher-dimensional generalizations of NLEEs and their associated soliton and rogue-wave dynamics. Several works have systematically explored a variety of (3 + 1)- and (4 + 1)-dimensional extensions, producing kink-solitons, solitary wave structures, and higher-order rogue waves across generalized KdV-, KP-, and P-type evolution equations [[5], [6], [7], [8]]. Further contributions have highlighted rogue-wave structures in nonlinear liquid–gas bubble systems [9] and dispersive solitons in novel higher-dimensional frameworks [10]. These studies collectively demonstrate both the mathematical richness and physical relevance of multi-dimensional soliton and rogue-wave theory in nonlinear sciences.

Another strand of progress has been methodological. Techniques such as Hirota’s bilinear method and symbolic computation have been systematically refined to provide more efficient ways of handling high-dimensional nonlinear models. Direct symbolic algorithms for Painlevé-integrable equations in plasmas have been proposed [11], while other works developed streamlined approaches to bilinearization in arbitrary dimensions [12] and symbolic exploration of soliton solutions in fluids and nonlinear sciences [13]. These methods are particularly relevant to the present study, where enhanced algebraic and modified sub-ODE approaches are employed in tandem with Jacobi and Weierstrass elliptic functions to extract soliton solutions.

To simulate complicated physical systems where derivative terms and higher-order nonlinear effects become important, a number of integrable and non-integrable extensions of the nonlinear Schrödinger equation (NLSE) have been developed [[14], [15], [16], [17], [18], [19], [20]]. The Kaup–Newell, Chen–Lee–Liu, and Gerjikov–Ivanov equations are examples of derivative NLSEs that have been important in explaining nonlinear pulse dynamics in dispersive media [[21], [22], [23]]. Motivated by the need for a more comprehensive framework, this section introduces a generalized concatenated model that encapsulates these well-known equations as special cases and extends their physical applicability through a unified formulation.

Three well-known equations exhibit derivative-type nonlinearities. The first is the Kaup–Newell (KN) equation:

(1) $$iq_{t}+aq_{xx}+ib{\left({\left|q\right|}^{2}q\right)}_{x}=0.$$

The second is the Chen–Lee–Liu (CLL) equation:

(2) $$iq_{t}+aq_{xx}+ib{\left|q\right|}^{2}q_{x}=0.$$

The last type is the Gerdjikov–Ivanov (GI) equation:

(3) $$iq_{t}+aq_{xx}+b{\left|q\right|}^{4}q+icq^{2}q_{x}^{*}=0,$$

where $q^{*}\left(x,t\right)$ denotes the complex conjugate of the function q(x,t), a,b,c are constants and $i=\sqrt{-1}$ (see [24]). Several generations and modifications of the NLSE have been proposed and studied [[24], [25], [26]].

In this work, we introduce a novel concatenated derivative NLSE model that unifies and extends the above-mentioned Eqs. (1)–(3) into a single comprehensive framework.

The new concatenation model is written for the first time as follows:

(4) $$\begin{array}{c} iq_{t}+aq_{xx}+i\left[c_{1}{\left|q\right|}^{2}q_{x}+c_{2}{\left({\left|q\right|}^{2}q\right)}_{x}\right]+c_{3}\left[ib_{1}q^{2}q_{x}^{*}+b_{2}{\left|q\right|}^{4}q\right]=0, \end{array}$$

where q(x,t) is a complex-valued function representing the wave profile, and $q^{*}\left(x,t\right)$ is its complex conjugate. The parameters $a,c_{1},c_{2},c_{3},b_{1}$ and $b_{2}$ are real constants, and $i^{2}=-1$.

The model (4) serves as a generalization of several known systems under specific parameter selections:

• When $c_{1}=c_{3}=0$, Eq. (4) reduces to the Kaup-Newell (KN) equation.
• When $c_{2}=c_{3}=0$, Eq. (4) reduces to the Chen-Lee-Liu (CLL) equation.
• When $c_{1}=c_{2}=0$, Eq. (4) reduces to the Gerjikov-Ivanov (GI) equation.

This concatenated model thus serves as a mathematical replica of incorporating the various aspects of Plasma Physics.

In this section, we perform a detailed mathematical analysis of the generalized concatenated derivative NLS model introduced in Eq. (4). To facilitate analytical treatment, we employ a traveling wave reduction by introducing a suitable ansatz that transforms the (PDE) into (ODE). This reduction enables the study of localized wave structures and soliton profiles under various parametric constraints. The resulting nonlinear ODE is then analyzed using appropriate mathematical techniques to extract exact solutions and explore their qualitative features.

To solve Eq. (4), set:

(5) q(x,t)=φ(ξ)expi[−κx+wt],ξ=x−Vt.

Here, κ, w, and V are real constants, whereV is the soliton velocity, w is the frequency, and κ is the wave number. The function φ(ξ) is real-valued. The function of ξ denotes the pulse’s form, with ξ serving as the traveling coordinate.

Substituting (5) into Eq. (4) and isolating the real and imaginary components yields two equations:

(6) $$a\varphi^{\prime \prime }\left(\xi \right)-[w+a\kappa^{2}]\varphi \left(\xi \right)+\kappa c_{1}+c_{2}-c_{3}b_{1}]\varphi^{3}\left(\xi \right)+c_{3}b_{2}\varphi^{5}\left(\xi \right)=0,$$

and

(7) $$-V-2a\kappa ]\varphi^{\prime }\left(\xi \right)+\left[c_{1}+3c_{2}+c_{3}b_{1}\right]\varphi^{\prime }\left(\xi \right)\varphi^{2}\left(\xi \right)=0.$$

Using the linear independence principle and Eq. (7), we derive:

(8) $$\begin{array}{c} V=-2a\kappa ,\\ c_{1}+3c_{2}+c_{3}b_{1}=0. \end{array}$$

By equating the highest derivative $\varphi^{\prime \prime }\left(\xi \right)$ with the nonlinear term $\varphi^{5}\left(\xi \right)$ in Eq. (6), the balance number is $N=\frac{1}{2}.$ However, according to the analytical methods described in the following section, the balance must be expressed as an integer. Setting

(9) $$\varphi \left(\xi \right)=\psi^{\frac{1}{2}}\left(\xi \right),$$

Eq. (6) changes to

(10) $${▵}_{1}\psi \left(\xi \right)\psi^{\prime \prime }\left(\xi \right)+{▵}_{2}\psi^{{}^{\prime }2}\left(\xi \right)+{▵}_{3}\psi^{2}\left(\xi \right)+{▵}_{4}\psi^{3}\left(\xi \right)+{▵}_{5}\psi^{4}\left(\xi \right)=0,$$

where

(11) $$\begin{array}{c} {▵}_{1}=\frac{a}{2},\\ {▵}_{2}=-\frac{a}{4},\\ {▵}_{3}=-\left(w+a\kappa^{2}\right),\\ {▵}_{4}=\kappa \left[c_{1}+c_{2}-c_{3}b_{1}\right],\\ {▵}_{5}=c_{3}b_{2}. \end{array}$$

In (6), the highest nonlinearity is quintic in φ. Setting $\varphi \left(\xi \right)=\psi^{1/2}\left(\xi \right)$ reduces the quintic nonlinearity to a quartic polynomial in ψ while preserving locality and positivity of physically relevant envelopes. Balancing $\psi \psi^{\prime \prime }$ with $\psi^{4}$ fixes the formal degree N=1 and leads to the reduced quartic form (10) with coefficients $\Delta_{1},\ldots ,\Delta_{5}$ in (11). This change of variables regularizes algebraic manipulations and aligns the reduced ODE with elliptic-function integrals, enabling the two solution routes developed below.

## Methods details

Zayed and Alngar developed a modified Sub-ODE approach [27], Zi-Liang-Li proposed a broader Sub-ODE methodology [28]. The additional approach to the modified Sub-ODE methodology, which was recently presented by Zayed et al. [29], will be used to solve Eq. (10). To do this, we assume that there is a formal solution to Eq. (10):

(12) $$\psi \left(\xi \right)=\sum_{s=0}^{N}\;A_{s}\;{\left[H\left(\xi \right)\right]}^{s},$$

where $A_{s}\left(s=0,1,2,..,N\right)$ are constants, provided $A_{N}\neq 0$, while H(ξ) is the solution of the auxiliary equation:

(13) $$H^{{}^{\prime }2}\left(\xi \right)=A\;H^{2-2p}\left(\xi \right)+B\;H^{2-p}\left(\xi \right)+C\;H^{2}\left(\xi \right)+DH^{2+p}\left(\xi \right)+E\;H^{2+2p}\left(\xi \right),$$

where A,B,C,D and E are constants, pis a positive integer. This section will employ the numerous specific solutions to Eq. (13), which are well known [27,29], to determine the soliton solutions of Eq. (10). IfD[ψ(ξ)]=N then $D\;[\psi^{{\;}^{\prime }}\left(\xi \right)]=N+p$, $D\;[\psi^{{\;}^{\prime \prime }}\left(\xi \right)]=N+2p$, thus $D\;[\psi^{\left(r\right)}\left(\xi \right)]=N+r\;p$ and $D\left[\psi^{\left(r\right)}\left(\xi \right)\;\psi^{s}\left(\xi \right)\right]=\left(s+1\right)N+pr$.

Balancing the highest derivative $\psi \left(\xi \right)\psi^{\prime \prime }\left(\xi \right)$ and nonlinear term $\psi^{4}\left(\xi \right)$ in Eq. (10), we have N+2P+N=4N⇒N=p.

**Case–1**: If, we pick out p=1, we have N=1, then the solution of Eq. (10) can be placed as follows:

(14) $$\psi \left(\xi \right)=A_{0}+A_{1}H\left(\xi \right)$$

where $A_{0},A_{1}$ are arbitrary real constants, $A_{1}\neq 0$, and H(ξ) satisfies the auxiliary equation

(15) $$H^{{}^{\prime }2}\left(\xi \right)=A\;+B\;H\left(\xi \right)+C\;H^{2}\left(\xi \right)+D\;H^{3}\left(\xi \right)+E\;H^{4}\left(\xi \right),\;E\neq 0.$$

Substituting Eqs. (14) and (15) into Eq. (10), and collecting all terms involving $\left[H{\left(\xi \right)}^{l}\right]{\left[H^{\prime }\left(\xi \right)\right]}^{f}$, (l=0,1,2,...4,F=0,1) we obtain a set of algebraic equations if we set these coefficients to zero:

(16) $$\begin{array}{c} H^{4}\left(\xi \right):\Delta_{5}A_{1}^{4}+\left(2\Delta_{1}+\Delta_{2}\right)EA_{1}^{2}=0,\\ H^{3}\left(\xi \right):\Delta_{2}A_{1}^{2}D+\frac{3}{2}\Delta_{1}A_{1}^{2}D+4\Delta_{5}A_{0}A_{1}^{3}+\Delta_{4}A_{1}^{3}+2\Delta_{1}A_{0}A_{1}E=0,\\ H^{2}\left(\xi \right):C\Delta_{1}A_{1}^{2}+C\Delta_{2}A_{1}^{2}+3\Delta_{4}A_{0}A_{1}^{2}+6\Delta_{5}A_{0}^{2}A_{1}^{2}+\frac{3}{2}D\Delta_{1}A_{0}A_{1}+\Delta_{3}A_{1}^{2}=0,\\ H\left(\xi \right):B\Delta_{2}A_{1}^{2}+\frac{1}{2}\Delta_{1}BA_{1}^{2}+2\Delta_{3}A_{0}A_{1}+3\Delta_{4}A_{0}^{2}A_{1}+4\Delta_{5}A_{0}^{3}A_{1}+C\Delta_{1}A_{0}A_{1}=0,\\ H^{0}\left(\xi \right):\frac{1}{2}B\Delta_{1}A_{1}A_{0}+\Delta_{2}A_{1}^{2}A+\Delta_{3}A_{0}^{2}+\Delta_{4}A_{0}^{3}+\Delta_{5}A_{0}^{4}=0. \end{array}\}$$

Now let’s examine the following sets:

**Set–1**: Using the Maple and inserting A=B=D=0 into the previously mentioned algebraic Eq. (16), we obtain:

(17) $$A_{0}=A_{0},\;A_{1}=\varepsilon {\left(-\frac{3E\Delta_{1}}{2\Delta_{5}}\right)}^{\frac{1}{2}},$$

and the constraint conditions

(18) $$\begin{array}{c} C=\frac{2\Delta_{5}A_{0}^{2}}{3\Delta_{1}},\\ \Delta_{2}=-\frac{\Delta_{1}}{2},\\ \Delta_{3}=\frac{5\Delta_{5}A_{0}^{2}}{3},\\ \Delta_{4}=\frac{5\Delta_{5}A_{0}^{2}}{3}, \end{array}$$

provided $E\Delta_{1}\Delta_{5}<0$ and ε=±1. With the aid of [27,29], we have the following solutions:

**(I)** If E<0and C>0, $\Delta_{1}\Delta_{5}>0$, we have the soliton solutions:

(19) $$q\left(x,t\right)=\varepsilon {\left[A_{0}\left(1+\;sech\;\left[\sqrt{\frac{2\Delta_{5}A_{0}^{2}}{3\Delta_{1}}}\;\left(x-Vt\right)\right]\right)\right]}^{\frac{1}{2}}e^{i\left(-\kappa x+wt\right)},$$

**(II)** If C>0, we have the exponential solution:

(20) $$q\left(x,t\right)=\varepsilon {\left[A_{0}+\sqrt{-\frac{3E\Delta_{1}}{2\Delta_{5}}}\frac{4Ce^{\varepsilon \sqrt{c}\left(x-Vt\right)}}{-1+4CEe^{2\varepsilon \sqrt{c}\left(x-Vt\right)}}\right]}^{\frac{1}{2}}e^{i\left(-\kappa x+wt\right)},$$

provided $A_{0}>0.$ The above two solutions (19) and (20) are obtained under the restrictions (18).

**Set–2**: Using the Maple and inserting B=D=0, $A=\frac{C^{2}}{4E},$ into the previously mentioned algebraic Eq. (16), we obtain:

(21) $$A_{0}=A_{0},\;A_{1}=A_{1},$$

and the constraint conditions

(22) $$\begin{array}{c} C=-\frac{2EA_{0}^{2}}{A_{1}^{2}},\\ \Delta_{1}=-\frac{\Delta_{5}A_{1}^{2}+E\Delta_{2}}{2E},\\ \Delta_{3}=-\frac{2A_{0}^{2}\left(-\Delta_{5}A_{1}^{2}+E\Delta_{2}\right)}{A_{1}^{2}},\\ \Delta_{4}=\frac{A_{0}\left(-3\Delta_{5}A_{1}^{2}+E\Delta_{2}\right)}{A_{1}^{2}}. \end{array}$$

With the aid of [27,29], we have the soliton solution:

(23) $$q\left(x,t\right)=\varepsilon {\left[A_{0}\left(1+\text{tanh}\left[\sqrt{\frac{EA_{0}^{2}}{A_{1}^{2}}}\;\left(x-Vt\right)\right]\right)\right]}^{\frac{1}{2}}e^{i\left(-\kappa x+wt\right)},$$

provided C〈0,E〉0, $A_{0}>0.$ The solution (23) is obtained under the restrictions (20).

**Set–3**: Using the Maple and inserting B=D=0, $A=\frac{e_{1}C^{2}}{E},$ into the previously mentioned algebraic Eq. (16), we obtain:

(24) $$A_{0}=A_{0},\;A_{1}=A_{1},$$

and the constraint conditions

(25) $$\begin{array}{c} C=-\frac{2EA_{0}^{2}}{A_{1}^{2}},\\ \Delta_{1}=-\frac{\Delta_{5}A_{1}^{2}+e_{1}E\Delta_{2}}{2E},\\ \Delta_{3}=-\frac{2A_{0}^{2}\left(-\Delta_{5}A_{1}^{2}+e_{1}E\Delta_{2}\right)}{A_{1}^{2}},\\ \Delta_{4}=\frac{A_{0}\left(-3\Delta_{5}A_{1}^{2}+e_{1}E\Delta_{2}\right)}{A_{1}^{2}}. \end{array}$$

These solutions are obtained with the help of [27,29]:

**(I)** When $e_{1}=\frac{m^{2}\left(m^{2}-1\right)}{{\left(2m^{2}-1\right)}^{2}}\;$then $A=\frac{e_{1}C^{2}}{E}$, 0<m<1, we have the Jacobi’s elliptic solution:

(26) $$q\left(x,t\right)=\varepsilon {\left[A_{0}+A_{1}\sqrt{-\frac{Cm^{2}}{E\left(2m^{2}-1\right)}}\;cn\;\left(\sqrt{\frac{C}{2m^{2}-1}}\left(x-Vt\right)\right)\right]}^{\frac{1}{2}}e^{i\left(-\kappa x+wt\right)},$$

provided $C(2m^{2}-1)>0,E<0.$

**(II)** When $e_{1}=\frac{\left(1-m^{2}\right)}{{\left(2-m^{2}\right)}^{2}}\;$then$\;A=\frac{e_{1}C^{2}}{E}$,0<m<1, we have the Jacobi’s elliptic solution:

(27) $$q\left(x,t\right)=\varepsilon {\left[A_{0}+A_{1}\sqrt{-\frac{C}{E\left(2-m^{2}\right)}}\;dn\;\left(\sqrt{\frac{C}{2-m^{2}}}\left(x-Vt\right)\right)\right]}^{\frac{1}{2}}e^{i\left(-\kappa x+wt\right)},$$

provided C>0,E<0. If $m\to 1^{-}$ in (26) and (27), we have the soliton solution:

(28) $$q\left(x,t\right)=\varepsilon {\left[A_{0}\left(1+\sqrt{2}\;sech\;\left(\sqrt{-\frac{2EA_{0}^{2}}{A_{1}^{2}}}\left(x-Vt\right)\right)\right)\right]}^{\frac{1}{2}}e^{i\left(-\kappa x+wt\right)},$$

provided $A_{0}>0.$

**(III)** When $e_{1}=\frac{m^{2}}{{\left(m^{2}+1\right)}^{2}}$ then $A=\frac{e_{1}C^{2}}{E}$, 0<m<1, we have the Jacobi’s elliptic solutions:

(29) $$q\left(x,t\right)=\varepsilon {\left[A_{0}+A_{1}\sqrt{-\frac{Cm^{2}}{E\left(m^{2}+1\right)}}\;sn\;\left(\sqrt{-\frac{C}{m^{2}+1}}\left(x-Vt\right)\right)\right]}^{\frac{1}{2}}e^{i\left(-\kappa x+wt\right)},$$

provide C<0 and E>0. If $m\to 1^{-}$ in (29), we have the soliton solution.

(30) $$q\left(x,t\right)=\varepsilon {\left[A_{0}+A_{1}\sqrt{-\frac{C}{2E}}\;tanh\;\left(\sqrt{-\frac{C}{2}}\left(x-Vt\right)\right)\right]}^{\frac{1}{2}}e^{i\left(-\kappa x+wt\right)},$$

All the solutions (26)-(30) are existed under the restrictions (25).

**Set–4**: Using the Maple and inserting B=D=0, into the previously mentioned algebraic Eq. (16), we obtain:

(31) $$A_{0}=A_{0},\;A_{1}=A_{1},$$

and the constraint conditions

(32) $$\begin{array}{c} A=-\frac{EA_{0}^{4}}{A_{1}^{4}},\;\\ C=-\frac{2EA_{0}^{2}}{A_{1}^{2}},\\ \Delta_{3}=-\frac{2EA_{0}^{2}\left(\Delta_{1}+\Delta_{2}\right)}{A_{1}^{2}},\\ \Delta_{4}=-\frac{2EA_{0}\left(3\Delta_{1}+2\Delta_{2}\right)}{A_{1}^{2}},\\ \Delta_{5}=-\frac{E\left(2\Delta_{1}+\Delta_{2}\right)}{A_{1}^{2}}. \end{array}$$

The following four solutions to Weierstrass elliptic functions are obtained with the help of [27,29]:

**(I)**

(33) $$q\left(x,t\right)=\varepsilon \;{\left[A_{0}+A_{1}{\left(\frac{1}{E}℘\left[\left(x-Vt\right),g_{2},g_{3}\right]-\frac{C}{3E}\right)}^{\frac{1}{2}}\right]}^{\frac{1}{2}}e^{i\left(-\kappa x+wt\right)},$$

**(II)**

(34) $$q\left(x,t\right)=\varepsilon {\left[A_{0}+A_{1}\;{\left(\frac{3A}{3℘\left[\left(x-Vt\right),g_{2},g_{3}\right]-C}\right)}^{\frac{1}{2}}\right]}^{\frac{1}{2}}e^{i\left(-\kappa x+wt\right)},$$

where the invariants $g_{2},g_{3}$ of the Weierstrass elliptic function solutions (33) and (34) are given by

(35) $$g_{2}=\frac{4C^{2}-12AE}{3}\;\text{and}\;g_{3}=\frac{4C\left(-2C^{2}+9AE\right)}{27}.$$

**(III)**

(36) $$q\left(x,t\right)=\varepsilon {\left[A_{0}+A_{1}\;\left(\frac{6\sqrt{A}℘\left[\left(x-Vt\right),g_{2},g_{3}\right]+C\sqrt{A}}{3{℘}^{\prime }\left[\left(x-Vt\right),g_{2},g_{3}\right]}\right)\right]}^{\frac{1}{2}}e^{i\left(-\kappa x+wt\right)},A>0.$$

**(IV)**

(37) $$q\left(x,t\right)=\varepsilon {\left[A_{0}+A_{1}\;\left(\frac{3\sqrt{\frac{1}{E}}{℘}^{\prime }\left[\left(x-Vt\right),g_{2},g_{3}\right]}{6℘\left[\left(x-Vt\right),g_{2},g_{3}\right]+C}\right)\right]}^{\frac{1}{2}}e^{i\left(-\kappa x+wt\right)},E>0.$$

where the Weierstrass elliptic function solutions (36), (37) have invariants $g_{2},g_{3}$ that are provided by

(38) $$g_{2}=\frac{C^{2}}{12}+AE\;\text{and}\;g_{3}=\frac{C\left(36AE-C^{2}\right)}{216}.$$

All the solutions (33), (34), (36) and (37) are obtained under the restrictions (32).

It is well known [30,31] that, the Weierstrass elliptic function $℘(\xi ;g_{2},g_{3})$ can be written as:

(39) $$\begin{array}{c} ℘\left(\xi ;g_{2},g_{3}\right)=l_{2}-\left(l_{2}-l_{3}\right)\;cn{\;}^{2}\left(\sqrt{l_{1}-l_{3}}\xi ;m\right),\\ ℘\left(\xi ;g_{2},g_{3}\right)=l_{3}+\left(l_{1}-l_{3}\right)\;ns{\;}^{2}\left(\sqrt{l_{1}-l_{3}}\xi ;m\right), \end{array}\}$$

in terms of the Jacobi’s elliptic functions where $m=\sqrt{\frac{l_{2}-l_{3}}{l_{1}-l_{3}}}$ is the modulus of the Jacobi’s elliptic function; $l_{j}\left(j=1,2,3\right)$, $l_{1}\geq l_{2}\geq l_{3}$ are the three roots of the cubic equation $4y^{3}-g_{2}y-g_{3}=0$.

To obtain Jacobi’s elliptic solutions, we substitute (39) into (33):

(40) $$q\left(x,t\right)=\varepsilon \;{\left[A_{0}+A_{1}{\left(\frac{1}{E}\left[l_{2}-\left(l_{2}-l_{3}\right)\;cn{\;}^{2}\left(\sqrt{l_{1}-l_{3}}\xi ;m\right)\right]-\frac{C}{3E}\right)}^{\frac{1}{2}}\right]}^{\frac{1}{2}}e^{i\left(-\kappa x+wt\right)},$$

and

(41) $$q\left(x,t\right)=\varepsilon \;{\left[A_{0}+A_{1}{\left(\frac{1}{E}\left[l_{3}+\left(l_{1}-l_{3}\right)\;ns{\;}^{2}\left(\sqrt{l_{1}-l_{3}}\xi ;m\right)\right]-\frac{C}{3E}\right)}^{\frac{1}{2}}\right]}^{\frac{1}{2}}e^{i\left(-\kappa x+wt\right)},$$

In particular, if m→1, then $l_{1}\to l_{2}$ and we have cn(ξ,1)→sech(ξ) and ns(ξ,1)→coth(ξ). Now, we have the soliton solutions:

(42) $$q\left(x,t\right)=\varepsilon \;{\left[A_{0}+A_{1}{\left(\frac{1}{E}\left[l_{2}-\left(l_{2}-l_{3}\right)\;sech{\;}^{2}\left(\sqrt{l_{2}-l_{3}}\xi ;m\right)\right]-\frac{C}{3E}\right)}^{\frac{1}{2}}\right]}^{\frac{1}{2}}e^{i\left(-\kappa x+wt\right)},$$

and the singular soliton solutions:

(43) $$q\left(x,t\right)=\varepsilon \;{\left[A_{0}+A_{1}{\left(\frac{1}{E}\left[l_{3}+\left(l_{2}-l_{3}\right)\;coth{\;}^{2}\left(\sqrt{l_{2}-l_{3}}\xi ;m\right)\right]-\frac{C}{3E}\right)}^{\frac{1}{2}}\right]}^{\frac{1}{2}}e^{i\left(-\kappa x+wt\right)},$$

Substituting (39) into (34) we have Jacobi’s elliptic solutions:

(44) $$q\left(x,t\right)=\varepsilon {\left[A_{0}+A_{1}\;{\left(\frac{A}{l_{2}-\left(l_{2}-l_{3}\right)\;cn{\;}^{2}\left(\sqrt{l_{1}-l_{3}}\xi ;m\right)-\frac{C}{3}}\right)}^{\frac{1}{2}}\right]}^{\frac{1}{2}}e^{i\left(-\kappa x+wt\right)},$$

and

(45) $$q\left(x,t\right)=\varepsilon {\left[A_{0}+A_{1}\;{\left(\frac{A}{l_{3}+\left(l_{1}-l_{3}\right)\;ns{\;}^{2}\left(\sqrt{l_{1}-l_{3}}\xi ;m\right)-\frac{C}{3}}\right)}^{\frac{1}{2}}\right]}^{\frac{1}{2}}e^{i\left(-\kappa x+wt\right)},$$

In particular, we have the soliton solutions:

(46) $$q\left(x,t\right)=\varepsilon {\left[A_{0}+A_{1}\;{\left(\frac{A}{l_{2}-\left(l_{2}-l_{3}\right)\;sech{\;}^{2}\left(\sqrt{l_{2}-l_{3}}\xi ;m\right)-\frac{C}{3}}\right)}^{\frac{1}{2}}\right]}^{\frac{1}{2}}e^{i\left(-\kappa x+wt\right)},$$

and

(47) $$q\left(x,t\right)=\varepsilon {\left[A_{0}+A_{1}\;{\left(\frac{A}{l_{3}+\left(l_{2}-l_{3}\right)\;coth{\;}^{2}\left(\sqrt{l_{2}-l_{3}}\xi ;m\right)-\frac{C}{3}}\right)}^{\frac{1}{2}}\right]}^{\frac{1}{2}}e^{i\left(-\kappa x+wt\right)},$$

Substituting (39) into (36) we have Jacobi’s elliptic solutions:

(48) $$q\left(x,t\right)=\varepsilon {\left[A_{0}+A_{1}\;\left(\frac{6\sqrt{A}\left[l_{2}-\left(l_{2}-l_{3}\right)\;cn{\;}^{2}\left(\sqrt{l_{1}-l_{3}}\xi ;m\right)\right]+C\sqrt{A}}{\sqrt{l_{1}-l_{3}}\left(l_{2}-l_{3}\right)\;cn\;\left(\sqrt{l_{1}-l_{3}}\xi ;m\right)\;sn\;\left(\sqrt{l_{1}-l_{3}}\xi ;m\right)\;dn\;\left(\sqrt{l_{1}-l_{3}}\xi ;m\right)}\right)\right]}^{\frac{1}{2}}e^{i\left(-\kappa x+wt\right)},$$

and

(49) $$q\left(x,t\right)=\varepsilon {\left[A_{0}+A_{1}\;\left(-\frac{6\sqrt{A}\left[l_{3}+\left(l_{1}-l_{3}\right)\;ns{\;}^{2}\left(\sqrt{l_{1}-l_{3}}\xi ;m\right)\right]+C\sqrt{A}}{\sqrt{l_{1}-l_{3}}\left(l_{1}-l_{3}\right)\;cn\;\left(\sqrt{l_{1}-l_{3}}\xi ;m\right)\;dn\;\left(\sqrt{l_{1}-l_{3}}\xi ;m\right)\;ns{\;}^{3}\left(\sqrt{l_{1}-l_{3}}\xi ;m\right)}\right)\right]}^{\frac{1}{2}}e^{i\left(-\kappa x+wt\right)},$$

where A>0.

In particular, we have the hybrid soliton solutions:

(50) $$q\left(x,t\right)=\varepsilon {\left[A_{0}+A_{1}\;\left(\frac{6\sqrt{A}\left[l_{2}-\left(l_{2}-l_{3}\right)\;sech{\;}^{2}\left(\sqrt{l_{2}-l_{3}}\xi ;m\right)\right]+C\sqrt{A}}{\sqrt{l_{2}-l_{3}}\left(l_{2}-l_{3}\right)\;sech{\;}^{2}\left(\sqrt{l_{2}-l_{3}}\xi ;m\right)\;tanh\;\left(\sqrt{l_{2}-l_{3}}\xi ;m\right)}\right)\right]}^{\frac{1}{2}}e^{i\left(-\kappa x+wt\right)},$$

and

(51) $$q\left(x,t\right)=\varepsilon {\left[A_{0}+A_{1}\;\left(-\frac{6\sqrt{A}\left[l_{3}+\left(l_{2}-l_{3}\right)\;coth{\;}^{2}\left(\sqrt{l_{2}-l_{3}}\xi ;m\right)\right]+C\sqrt{A}}{\sqrt{l_{2}-l_{3}}\left(l_{2}-l_{3}\right)\;sech{\;}^{2}\left(\sqrt{l_{2}-l_{3}}\xi ;m\right)\;coth{\;}^{3}\left(\sqrt{l_{2}-l_{3}}\xi ;m\right)}\right)\right]}^{\frac{1}{2}}e^{i\left(-\kappa x+wt\right)},$$

provided A>0.

Jacobi’s elliptic solutions can be obtained by substituting (39) into (37):

(52) $$q\left(x,t\right)=\varepsilon {\left[A_{0}+A_{1}\;\left(\frac{3\sqrt{\frac{1}{E}}\sqrt{l_{1}-l_{3}}\left(l_{2}-l_{3}\right)\;cn\;\left(\sqrt{l_{1}-l_{3}}\xi ;m\right)\;sn\;\left(\sqrt{l_{1}-l_{3}}\xi ;m\right)\;dn\;\left(\sqrt{l_{1}-l_{3}}\xi ;m\right)}{6\left[l_{2}-\left(l_{2}-l_{3}\right)\;cn{\;}^{2}\left(\sqrt{l_{1}-l_{3}}\xi ;m\right)\right]+C}\right)\right]}^{\frac{1}{2}}e^{i\left(-\kappa x+wt\right)},$$

and

(53) $$q\left(x,t\right)=\varepsilon {\left[A_{0}+A_{1}\;\left(-\frac{3\sqrt{\frac{1}{E}}\sqrt{l_{1}-l_{3}}\left(l_{1}-l_{3}\right)\;cn\;\left(\sqrt{l_{1}-l_{3}}\xi ;m\right)\;dn\;\left(\sqrt{l_{1}-l_{3}}\xi ;m\right)\;ns{\;}^{3}\left(\sqrt{l_{1}-l_{3}}\xi ;m\right)}{6\left[l_{3}+\left(l_{1}-l_{3}\right)\;ns{\;}^{2}\left(\sqrt{l_{1}-l_{3}}\xi ;m\right)\right]+C}\right)\right]}^{\frac{1}{2}}e^{i\left(-\kappa x+wt\right)},$$

provided E>0. In particular, we have the straddled soliton solutions:

(54) $$q\left(x,t\right)=\varepsilon {\left[A_{0}+A_{1}\;\left(\frac{3\sqrt{\frac{1}{E}}\sqrt{l_{2}-l_{3}}\left(l_{2}-l_{3}\right)\;sech{\;}^{2}\left(\sqrt{l_{2}-l_{3}}\xi ;m\right)\;tanh\;\left(\sqrt{l_{2}-l_{3}}\xi ;m\right)}{6\left[l_{2}-\left(l_{2}-l_{3}\right)\;sech{\;}^{2}\left(\sqrt{l_{2}-l_{3}}\xi ;m\right)\right]+C}\right)\right]}^{\frac{1}{2}}e^{i\left(-\kappa x+wt\right)},$$

and

(55) $$q\left(x,t\right)=\varepsilon {\left[A_{0}+A_{1}\;\left(-\frac{3\sqrt{\frac{1}{E}}\sqrt{l_{2}-l_{3}}\left(l_{2}-l_{3}\right)\;sech{\;}^{2}\left(\sqrt{l_{2}-l_{3}}\xi ;m\right)\;coth{\;}^{3}\left(\sqrt{l_{2}-l_{3}}\xi ;m\right)}{6\left[l_{3}+\left(l_{2}-l_{3}\right)\;coth{\;}^{2}\left(\sqrt{l_{2}-l_{3}}\xi ;m\right)\right]+C}\right)\right]}^{\frac{1}{2}}e^{i\left(-\kappa x+wt\right)},$$

where E>0. All the above solutions (40–55) are obtained under the restrictions (32).

**Set–5**: Using the Maple and inserting B=D=0, $A=\frac{5C^{2}}{36E},$ into the previously mentioned algebraic Eq. (16), we obtain:

(56) $$A_{0}=A_{0},\;A_{1}=\varepsilon {\left(-\frac{3E\Delta_{2}}{\Delta_{5}}\right)}^{\frac{1}{2}},$$

and

(57) $$\begin{array}{c} C=\frac{2\Delta_{5}A_{0}^{2}}{5\Delta_{2}},\\ \Delta_{1}=-2\Delta_{2},\\ \Delta_{3}=-\frac{8A_{0}^{2}}{5\Delta_{5}},\\ \Delta_{4}=\frac{8\Delta_{5}A_{0}}{3}, \end{array}$$

provided $E\Delta_{2}\Delta_{5}\;<0,\varepsilon =\pm 1$. The following solution to Weierstrass elliptic functions are obtained with the help of [27,29]:

(58) $$q\left(x,t\right)=\varepsilon {\left[A_{0}+A_{1}\;\left(\frac{\sqrt{\frac{5C^{2}}{E}}℘\left[\left(x-Vt\right),g_{2},g_{3}\right]+\frac{C}{6}}{18{℘}^{\prime }\left[\left(x-Vt\right),g_{2},g_{3}\right]}\right)\right]}^{\frac{1}{2}}e^{i\left(-\kappa x+wt\right)},$$

where the invariants $g_{2},g_{3}$ of the Weierstrass elliptic function solution (58) are given by

(59) $$g_{2}=\frac{2C^{2}}{9}\;\text{and}\;g_{3}=\frac{C^{3}}{54}.$$

The solution (58) exists under the restrictions given in Eq. (57).

**Set–6**: Using the Maple and inserting A=B=0, into the previously mentioned algebraic Eq. (16), we obtain:

(60) $$A_{0}=0,\;A_{1}=\;A_{1},$$

and the constraint conditions

(61) $$\begin{array}{c} \Delta_{3}=-C\left(\Delta_{1}+\Delta_{2}\right),\\ \Delta_{4}=-\frac{D\left(3\Delta_{1}+2\Delta_{2}\right)}{A_{1}},\\ \Delta_{5}=-\frac{4C^{2}\left(2\Delta_{1}+\Delta_{2}\right)-D^{2}\left(2\Delta_{1}+\Delta_{2}\right)}{4CA_{1}^{2}}, \end{array}$$

With the aid of [27,29], we have the soliton solutions:

(62) $$q\left(x,t\right)=\varepsilon {\left[\frac{A_{1}}{\text{cosh}\sqrt{C}\;\left(x-Vt\right)-\frac{D}{2C}}\right]}^{\frac{1}{2}}e^{i\left(-\kappa x+wt\right)},$$

provided $A_{1}>0.$ The solution (62) is existed under the restrictions (61).

**Set–7**: Using the Maple and inserting $A=B=0,D=-2\sqrt{CE}$, into the previously mentioned algebraic Eq. (16), we obtain:

(63) $$A_{0}=0,\;A_{1}=\;A_{1},$$

and the constraint conditions

(64) $$\begin{array}{c} \Delta_{1}=\frac{A_{1}\left(2\sqrt{CE}\Delta_{5}A_{1}+E\Delta_{4}\right)}{E\sqrt{CE}},\\ \Delta_{2}=-\frac{A_{1}\left(3\sqrt{CE}\Delta_{5}A_{1}+2E\Delta_{4}\right)}{E\sqrt{CE}},\\ \Delta_{3}=-\frac{CA_{1}\left(\sqrt{CE}\Delta_{5}A_{1}+E\Delta_{4}\right)}{E\sqrt{CE}}\;. \end{array}$$

With the aid of [27,29], we have the soliton solutions:

(65) $$q\left(x,t\right)=\varepsilon {\left[\frac{A_{1}}{2}\sqrt{\frac{C}{E}}\left(1+\frac{1}{2}\text{tanh}\sqrt{C}\;\left(x-Vt\right)\right)\right]}^{\frac{1}{2}}e^{i\left(-\kappa x+wt\right)},$$

provided $A_{1}>0.$ The solution (65) is existed under the restrictions (64).

By varying the values of the parameters for p and N, it is also possible to generate many soliton solutions of Eq. (10).

Eq. (10) has another formal solution based on the enhanced direct algebraic method [[32], [33], [34]]:

(66) $$\psi \left(\xi \right)=\alpha_{0}+\sum_{i=1}^{N}\;\left\{\alpha_{i}\;F^{i}\left(\xi \right)+\beta_{i}\;F^{-i}\left(\xi \right)\right\},$$

provided $\alpha_{N}^{2}+\beta_{N}^{2}\;\neq 0$, while F(ξ) is the solution of the equation:

(67) $$F^{{\;}^{\prime }2}\left(\xi \right)=\sum_{l=0}^{4}\;L_{l}\;F^{l}\left(\xi \right),$$

where $L_{j}\left(j=0,1,2,3,4\right)$ are constants, provided $L_{4}\neq 0$. Balancing $\psi \left(\xi \right)\psi^{\prime \prime }\left(\xi \right)$ and nonlinear term $\psi^{4}\left(\xi \right)$ in Eq. (10), we get the balance number N=1. Now, Eq. (10) has the formal solution:

(68) $$\psi \left(\xi \right)=\alpha_{0}+\alpha_{1}F\left(\xi \right)+\frac{\beta_{1}}{F\left(\xi \right)},$$

where $\alpha_{0},\alpha_{1}$ and $\beta_{1}$ are constants to be determined, provided $\alpha_{1}^{2}+\beta_{1}^{2}\;\neq 0$. The following system of algebraic equations is obtained by substituting (68) and Eq. (67) into Eq. (10) and setting all of the coefficients of $F^{i}\left(\xi \right){\left(F^{\prime }\left(\xi \right)\right)}^{j},\left(i=-4,...,-1,0,1,2,...,4,j=0,1\right)$ to zero,

(69) $$\begin{array}{c} F^{4}\left(\xi \right):{▵}_{5}\alpha_{1}^{4}+\alpha_{1}^{2}L_{4}\left(2{▵}_{1}+{▵}_{2}\right)=0,\\ F^{3}\left(\xi \right):2{▵}_{1}\alpha_{0}\alpha_{1}L_{4}+\alpha_{1}^{2}L_{3}\left(\frac{3}{2}{▵}_{1}+{▵}_{2}\right)+\alpha_{1}^{3}\left({▵}_{4}+4{▵}_{5}\alpha_{0}\right)=0,\\ F^{2}\left(\xi \right):\frac{3}{2}{▵}_{1}\alpha_{0}\alpha_{1}L_{3}+2\alpha_{1}L_{4}\beta_{1}\left({▵}_{1}-{▵}_{2}\right)+\alpha_{1}^{2}L_{2}\left({▵}_{1}+{▵}_{2}\right)\\ \;+\alpha_{1}^{2}\left(4{▵}_{4}\alpha_{0}+6{▵}_{5}\alpha_{0}^{2}+4{▵}_{5}\beta_{1}\right)=0,\\ F\left(\xi \right):\alpha_{0}\alpha_{1}\left({▵}_{1}L_{2}+2{▵}_{3}\right)+2\alpha_{1}L_{3}\beta_{1}\left({▵}_{1}-{▵}_{2}\right)+\alpha_{1}^{2}L_{1}\left(\frac{1}{2}{▵}_{1}+{▵}_{2}\right)\\ +\alpha_{1}\left(3{▵}_{4}\alpha_{0}^{2}+3{▵}_{4}\alpha_{1}\beta_{1}+15{▵}_{5}\alpha_{0}\alpha_{1}\beta_{1}+4{▵}_{5}\alpha_{0}^{3}\right)=0,\\ F^{-4}\left(\xi \right):{▵}_{5}\beta_{1}^{4}+\beta_{1}^{2}L_{0}\left(2{▵}_{1}+{▵}_{2}\right)=0,\\ F^{-3}\left(\xi \right):2{▵}_{1}\alpha_{0}\beta_{1}L_{0}+\beta_{1}^{2}L_{1}\left(\frac{3}{2}{▵}_{1}+{▵}_{2}\right)+\beta_{1}^{3}\left({▵}_{4}+4{▵}_{5}\alpha_{0}\right)=0,\\ F^{-2}\left(\xi \right):\frac{3}{2}{▵}_{1}\alpha_{0}\beta_{1}L_{1}+2\alpha_{1}L_{0}\beta_{1}\left({▵}_{1}-{▵}_{2}\right)+\beta_{1}^{2}L_{2}\left({▵}_{1}+{▵}_{2}\right)\\ \;+\beta_{1}^{2}\left(3{▵}_{4}\alpha_{0}+6{▵}_{5}\alpha_{0}^{2}+4{▵}_{5}\alpha_{1}+{▵}_{3}\right)=0,\\ F^{-1}\left(\xi \right):{▵}_{1}\alpha_{0}\beta_{1}L_{2}+L_{3}\beta_{1}^{3}\left(\frac{1}{2}{▵}_{1}+{▵}_{2}\right)+2\alpha_{1}L_{1}\beta_{1}\left({▵}_{1}-{▵}_{2}\right)\\ +\beta_{1}\left(3{▵}_{4}\alpha_{0}^{2}+3{▵}_{4}\alpha_{1}\beta_{1}+15{▵}_{5}\alpha_{0}\alpha_{1}\beta_{1}+4{▵}_{5}\alpha_{0}^{3}+2{▵}_{3}\alpha_{0}\right)=0,\\ F^{0}\left(\xi \right):2\alpha_{1}L_{2}\beta_{1}\left({▵}_{1}-{▵}_{2}\right)+6\alpha_{1}\beta_{1}\left({▵}_{4}+2{▵}_{5}\alpha_{0}\right)+{▵}_{2}\left(\beta_{1}^{2}L_{4}+\alpha_{1}^{2}L_{0}\right)\\ +\frac{1}{2}{▵}_{1}\left(\alpha_{0}\beta_{1}L_{3}+\alpha_{0}\alpha_{1}L_{1}\right)+6{▵}_{5}\alpha_{1}^{2}\beta_{1}^{2}+2{▵}_{3}\alpha_{1}\beta_{1}+{▵}_{5}\alpha_{0}^{4}\\ +{▵}_{4}\alpha_{0}^{3}+{▵}_{3}\alpha_{0}^{2}=0. \end{array}\}$$

Now, let us discuss the following cases for the algebraic Eq. (69):

**Case–1**: Using the Maple, put $L_{0}=L_{1}=L_{3}=0,$ in the Eqs. (69), we have the results

(70) $$\alpha_{0}=\sqrt{\frac{3{▵}_{3}}{5{▵}_{5}}},\;\beta_{1}=0,\;\alpha_{1}=\sqrt{\frac{3{▵}_{1}L_{4}}{2{▵}_{5}}},\;L_{2}=\frac{2{▵}_{3}}{5{▵}_{1}}$$

with constraint conditions:

(71) $$\begin{array}{c} {▵}_{2}=-\frac{{▵}_{1}}{2},\\ {▵}_{4}=-\frac{8}{15}\sqrt{15{▵}_{3}{▵}_{5}}, \end{array}$$

provided ${▵}_{3}{▵}_{5}>0,{▵}_{1}{▵}_{5}L_{4}>0$.

When $L_{2}>0,\;{▵}_{1}{▵}_{3}>0$ and $L_{4}<0$, ${▵}_{1}{▵}_{5}<0$ . Eq. (4) has the bell-shaped soliton solutions:

(72) $$q\left(x,t\right)={\left[\sqrt{\frac{3{▵}_{3}}{5{▵}_{5}}}\left(1+\;sech\;\left[\sqrt{\frac{2{▵}_{3}}{5{▵}_{1}}}\left(x-Vt\right)\;\right]\right)\right]}^{\frac{1}{2}}e^{i\left(-\kappa x+wt\right)},$$

where ${▵}_{3}{▵}_{5}>0$.

Also, Eq. (4) has singular soliton solutions when $L_{2}>0,\;{▵}_{1}{▵}_{3}>0\;$and $L_{4}>0,\;{▵}_{1}{▵}_{5}>0$ as the following:

(73) $$q\left(x,t\right)={\left[\sqrt{\frac{3{▵}_{3}}{5{▵}_{5}}}\left(1+\;csch\;\left[\sqrt{\frac{2{▵}_{3}}{5{▵}_{1}}}\left(x-Vt\right)\;\right]\right)\right]}^{\frac{1}{2}}e^{i\left(-\kappa x+wt\right)},$$

where ${▵}_{3}{▵}_{5}>0$. Constraint conditions (71) exist for the solutions (72,73).

Fig. 1, 2

**Fig. 1:**
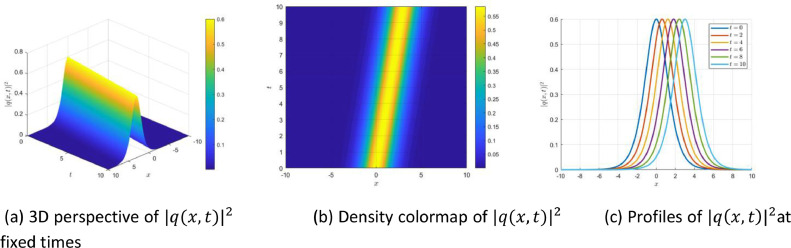
The dynamics of the localized envelope soliton, as derived from Eq. (72), are illustrated. (a) A three-dimensional plot of $|q\left(x,t\right){|}^{2}$, (b) a heatmap depicting its propagation; and (c) temporal profiles illustrating the soliton’s bell-shaped, stable structure.

**Fig. 2:**
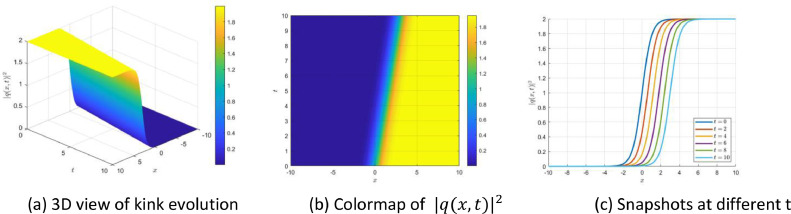
The kink-type soliton solution is obtained from Eq. (76). The panels show the evolution of its profile through three-dimensional, density, and time-sliced visualizations.

**Case–2**: Using the Maple, put $L_{0}=\frac{L_{2}^{2}}{4L_{4}},L_{1}=L_{3}=0,$ the Eqs. (69), we have the results

(74) $$\alpha_{0}=\alpha_{0},\;\beta_{1}=0,\;\alpha_{1}=\alpha_{1},\;L_{2}=-\frac{2\alpha_{0}^{2}L_{4}}{\alpha_{1}^{2}},$$

with constraint conditions:

(75) $$\begin{array}{c} {▵}_{1}=-\frac{\alpha_{1}^{2}{▵}_{5}+{▵}_{2}L_{4}}{2L_{4}},\\ {▵}_{3}=-\frac{2\alpha_{0}^{2}\left(-\alpha_{1}^{2}{▵}_{5}+{▵}_{2}L_{4}\right)}{\alpha_{1}^{2}},\\ {▵}_{4}=\frac{\alpha_{0}\left(-3\alpha_{1}^{2}{▵}_{5}+{▵}_{2}L_{4}\right)}{\alpha_{1}^{2}}, \end{array}$$

provided $L_{4}\neq 0$. When $L_{2}<0\;$and $L_{4}>0$, Eq. (4) has the kink-shaped soliton solutions:

(76) $$q\left(x,t\right)={\left[\alpha_{0}\left(1+\;tanh\;\left[\sqrt{\frac{\alpha_{0}^{2}L_{4}}{\alpha_{1}^{2}}}\left(x-Vt\right)\;\right]\right)\right]}^{\frac{1}{2}}e^{i\left(-\kappa x+wt\right)},$$

Also, Eq. (4) has singular soliton solutions as the following:

(77) $$q\left(x,t\right)={\left[\alpha_{0}\left(1+\;coth\;\left[\sqrt{\frac{\alpha_{0}^{2}L_{4}}{\alpha_{1}^{2}}}\left(x-Vt\right)\;\right]\right)\right]}^{\frac{1}{2}}e^{i\left(-\kappa x+wt\right)}.$$

provided $\alpha_{0}>0$. Under the constraints of (75), the solutions (76, 77) exist.

**Case–3**: Using the Maple, put $L_{1}=L_{3}=0,$ the Eqs. (69), we have the results:

**(I)** When $L_{0}=\frac{m^{2}\left(1-m^{2}\right)L_{2}}{\left(2m^{2}-1\right)L_{4}}$, 0<m<1,

(78) $$\begin{array}{c} \alpha_{0}=\alpha_{0},\;\beta_{1}=0,\\ \alpha_{1}=\frac{\sqrt{L_{2}L_{4}}}{2mL_{2}\sqrt{m^{2}-1}}{\left(4m^{4}-4m^{2}+1+\alpha_{0}\sqrt{32m^{8}-64m^{6}+44m^{4}-12m^{2}+1}\right)}^{\frac{1}{2}}, \end{array}$$

with constraint conditions:

(79) $$\begin{array}{c} {▵}_{1}=-\frac{L_{2}L_{4}}{3m^{2}L_{2}^{2}L_{4}\left(m^{2}-1\right)}\left(4m^{4}-4m^{2}+1+\alpha_{0}^{2}{▵}_{5}\sqrt{32m^{8}-64m^{6}+44m^{4}-12m^{2}+1}\right),\\ {▵}_{2}=-\frac{{▵}_{1}}{2},\\ {▵}_{3}=\frac{\alpha_{0}^{2}{▵}_{5}}{3L_{4}}\left(\frac{L_{2}L\left(4m^{4}-4m^{2}+1\right)}{2m^{2}L_{2}\left(m^{2}-1\right)}+\sqrt{32m^{8}-64m^{6}+44m^{4}-12m^{2}+1}+6L_{4}\right),\\ {▵}_{4}=-\frac{8}{3}\alpha_{0}{▵}_{5}. \end{array}$$

Eq. (4) has the Jacobi’s elliptic function solutions:

(80) $$q\left(x,t\right)=\varepsilon {\left[\alpha_{0}+\alpha_{1}\;cn\;\left(\sqrt{\frac{L_{2}}{2m^{2}-1}}\left(x-Vt\right),m\right)\right]}^{\frac{1}{2}}e^{i\left(-\kappa x+wt\right)},$$

provided $\left(2m^{2}-1\right)L_{2}>0.$In particular when m→1 in (80), we have the bell-shaped soliton

(81) $$q\left(x,t\right)=\varepsilon {\left[\alpha_{0}+\alpha_{1}\;sech\;\left(\sqrt{L_{2}}\left(x-Vt\right)\right)\right]}^{\frac{1}{2}}e^{i\left(-\kappa x+wt\right)}.$$

provided $L_{2}>0.$ Constraint conditions (79) exist for the solutions (80), (81).

**(II)** When $L_{0}=\frac{\left(1-m^{2}\right)L_{2}^{2}}{{\left(2-m^{2}\right)}^{2}L_{4}},\;0<m<1,$

(82) $$\alpha_{0}=\sqrt{\frac{L_{4}\left(m^{2}-2\right)}{L_{2}\left(m^{2}-1\right)}}\beta_{1},\;\beta_{1}=\beta_{1},\alpha_{1}=0,$$

with constraint conditions:

(83) $$\begin{array}{c} {▵}_{1}=\frac{2\beta_{1}^{2}L_{4}{▵}_{5}\left(m^{4}-4m^{2}+4\right)}{3L_{2}^{2}\left(m^{2}-1\right)},\\ {▵}_{2}=-\frac{{▵}_{1}}{2},\\ {▵}_{3}=-\frac{2\beta_{1}^{2}L_{4}{▵}_{5}\left(m^{4}+2m^{2}-8\right)}{3L_{2}\left(m^{2}-1\right)},\\ {▵}_{4}=-\frac{8\beta_{1}L_{4}{▵}_{5}\left(m^{2}-2\right)}{3\sqrt{\frac{L_{4}\left(m^{2}-2\right)}{L_{2}\left(m^{2}-1\right)}}L_{2}\left(m^{2}-1\right)}. \end{array}$$

Eq. (4) has the Jacobi’s elliptic function solutions:

(84) $$q\left(x,t\right)=\varepsilon {\left[\beta_{1}\left(\sqrt{\frac{L_{4}\left(2-m^{2}\right)}{L_{2}\left(m^{2}-1\right)}}+\frac{1}{\sqrt{-\frac{m^{2}L_{2}}{\left(2-m^{2}\right)L_{4}}}\;dn\;\left(\sqrt{\frac{L_{2}}{2-m^{2}}}\left(x-Vt\right),m\right)}\right)\right]}^{\frac{1}{2}}e^{i\left(-\kappa x+wt\right)},$$

provided $\beta_{1}>0,\left(2-m^{2}\right)L_{2}>0,L_{4}<0.$ Given the constraints (83), the solution (84) exists.

**(III)** When $L_{0}=\frac{m^{2}L_{2}^{2}}{{\left(m^{2}+1\right)}^{2}L_{4}}$, 0<m<1,

(85) $$\alpha_{0}=\sqrt{-\frac{L_{4}\left(m^{2}+1\right)}{L_{2}}}\beta_{1},\;\beta_{1}=\beta_{1},\alpha_{1}=0,$$

with constraint conditions:

(86) $$\begin{array}{c} {▵}_{1}=-\frac{2\beta_{1}^{2}L_{4}{▵}_{5}\left(m^{4}+2m^{2}+1\right)}{3L_{2}^{2}m^{2}},\\ {▵}_{2}=-\frac{{▵}_{1}}{2},\\ {▵}_{3}=-\frac{\beta_{1}^{2}L_{4}{▵}_{5}\left(5m^{4}+4m^{2}-1\right)}{3L_{2}m^{2}},\\ {▵}_{4}=-\frac{8\beta_{1}{▵}_{5}}{3}\sqrt{-\frac{L_{4}\left(m^{2}+1\right)}{L_{2}}}. \end{array}$$

Eq. (4) has the Jacobi’s elliptic functions:

(87) $$q\left(x,t\right)=\varepsilon {\left[\beta_{1}\left(\sqrt{-\frac{L_{4}\left(m^{2}+1\right)}{L_{2}}}+\frac{1}{\sqrt{-\frac{m^{2}L_{2}}{\left(m^{2}+1\right)L_{4}}}\;sn\;\left(\sqrt{-\frac{L_{2}}{m^{2}+1}}\left(x-Vt\right),m\right)}\right)\right]}^{\frac{1}{2}}e^{i\left(-\kappa x+wt\right)},$$

provided $L_{2}<0,L_{4}$ $>0,\beta_{1}>0.$In particular when m→1,we have the kink-shaped soliton solution.

(88) $$q\left(x,t\right)=\varepsilon {\left[\beta_{1}\left(\sqrt{-\frac{2L_{4}}{L_{2}}}+\frac{1}{\sqrt{-\frac{L_{2}}{2L_{4}}}\;sn\;\left(\sqrt{-\frac{L_{2}}{2}}\left(x-Vt\right)\right)}\right)\right]}^{\frac{1}{2}}e^{i\left(-\kappa x+wt\right)},$$

where $L_{2}\langle 0,L_{4}\rangle 0,\beta_{1}>0.$ The solutions (87), (88) are exist under constraint conditions (86).

**Case–4**: Using the Maple, put $L_{1}=L_{3}=0,$ the Eqs. (69), we have the results

(89) $$\alpha_{0}=\alpha_{0},\;\beta_{1}=\beta_{1},\;\alpha_{1}=0,\;L_{0}=-\frac{\beta_{1}^{2}L_{2}}{2\alpha_{0}^{2}},\;L_{4}=-\frac{\alpha_{0}^{2}L_{2}}{2\beta_{1}^{2}}$$

with constraint conditions:

(90) $$\begin{array}{c} {▵}_{3}=2L_{2}\left({▵}_{1}+{▵}_{2}\right),\\ {▵}_{4}=-\frac{L_{2}\left(3{▵}_{1}+2{▵}_{2}\right)}{\alpha_{0}},\\ {▵}_{5}=\frac{L_{2}\left(2{▵}_{1}+{▵}_{2}\right)}{2\alpha_{0}^{2}}. \end{array}$$

Eq. (4) has the Weierstrass elliptic doubly periodic type solution:

(91) $$q\left(x,t\right)=\varepsilon {\left[\alpha_{0}\left(1+\sqrt{-\frac{L_{2}}{2}}\frac{6℘\left[\left(x-Vt\right),g_{2},g_{3}\right]+L_{2}}{3{℘}^{\prime }\left[\left(x-Vt\right),g_{2},g_{3}\right]}\right)\right]}^{\frac{1}{2}}e^{i\left(-\kappa x+wt\right)},$$

where $\alpha_{0}>0,L_{2}<0.$ where the invariants $g_{2},g_{3}$ of the Weierstrass elliptic function solutions (91) is given by

(92) $$g_{2}=\frac{L_{2}^{2}}{12}+L_{0}L_{4}\;\text{and}\;g_{3}=\frac{L_{2}\left(36L_{0}L_{4}-L_{2}^{2}\right)}{216}.$$

Given the constraint constraints (90), the solution (91) is obtained.

**Case–5**: Using the Maple, put $L_{0}=$ $L_{1}=0,$the Eq.(69), we have the results;

(93) $$\alpha_{0}=0,\;\beta_{1}=0,\alpha_{1}=\alpha_{1},$$

with constraint conditions

(94) $$\begin{array}{c} {▵}_{3}=-L_{2}\left({▵}_{1}+{▵}_{2}\right),\\ {▵}_{4}=-\frac{L_{3}\left(3{▵}_{1}+2{▵}_{2}\right)}{2\alpha_{1}},\\ {▵}_{5}=-\frac{L_{4}\left(2{▵}_{1}+{▵}_{2}\right)}{\alpha_{1}^{2}}, \end{array}$$

provided $\alpha_{1}\neq 0$. Eq. (4) has the straddled soliton solutions as the following:

**(I)** when $L_{2}>0$ and $L_{4}>0$

(95) $$q\left(x,t\right)=\varepsilon {\left[\frac{\alpha_{1}L_{2}sech^{2}\left(\frac{\sqrt{L_{2}}}{2}\left(x-Vt\right)\right)}{\pm 2\sqrt{L_{2}L_{4}}\text{tanh}\left(\frac{\sqrt{L_{2}}}{2}\;\left(x-Vt\right)\right)+L_{3}}\right]}^{\frac{1}{2}}e^{i\left(-\kappa x+wt\right)},$$

(96) $$q\left(x,t\right)=\varepsilon {\left[\frac{\alpha_{1}L_{2}\text{csc}h^{2}\;\left(\frac{\sqrt{L_{2}}}{2}\left(x-Vt\right)\right)}{\pm 2\sqrt{L_{2}L_{4}}\text{coth}\left(\frac{\sqrt{L_{2}}}{2}\left(x-Vt\right)\right)+L_{3}}\right]}^{\frac{1}{2}}e^{i\left(-\kappa x+wt\right)},$$

where $\alpha_{1}L_{2}>0$ and $L_{2}L_{4}>0.$

**(II)** when $L_{2}>0$ and $L_{3}\neq 0$

(97) $$q\left(x,t\right)=\varepsilon {\left[\frac{\alpha_{1}L_{2}L_{3}sech^{2}\;\left(\frac{\sqrt{L_{2}}}{2}\left(x-Vt\right)\right)}{L_{3}^{2}-L_{2}L_{4}{\left[1-\text{tanh}\left(\frac{\sqrt{L_{2}}}{2}\;\left(x-Vt\right)\right)\right]}^{2}}\right]}^{\frac{1}{2}}e^{i\left(-\kappa x+wt\right)},$$

(98) $$q\left(x,t\right)=\varepsilon {\left[\frac{\alpha_{1}L_{2}L_{3}\text{csc}h^{2}\;\left(\frac{\sqrt{L_{2}}}{2}\left(x-Vt\right)\right)}{L_{3}^{2}-L_{2}L_{4}{\left[1-\text{coth}\left(\frac{\sqrt{L_{2}}}{2}\;\left(x-Vt\right)\right)\right]}^{2}}\right]}^{\frac{1}{2}}e^{i\left(-\kappa x+wt\right)},$$

provided $L_{2}>0,\alpha_{1}L_{3}>0.$ Under the constraint constraints (94), all of the solutions (95–98) are attained.

## Method validation

In this work, we introduced a novel concatenated derivative nonlinear Schrödinger (DNLS) equation model that unifies the Kaup–Newell, Chen–Lee–Liu, and Gerdjikov–Ivanov equations. This generalized framework encapsulates a wide range of plasma wave phenomena, including Langmuir waves, Alfvén waves, and cold plasma dynamics.

By applying a traveling wave reduction, the original partial differential equation (PDE) was reduced to a nonlinear ODE, which served as the basis for constructing exact soliton solutions. Two robust integrability techniques—the modified sub-ODE approach and the enhanced direct algebraic method—were employed to derive a broad class of analytical solutions expressed in terms of Weierstrass and Jacobi elliptic functions.

The obtained solutions can be broadly categorized based on their structure and physical implications. Bell-shaped solitons, such as those in Eqs. (19), (72), and (81), exhibit *Sech*-type profiles and represent stable, localized wave packets that preserve their shape during propagation. Kink and anti-kink solitons, appearing in Eqs. (23), (76), and (88), possess tanh-type profiles that model domain-wall-like transitions between different plasma states. Periodic and quasi-periodic solutions based on elliptic functions as in Eqs. (26)–(30), (40)–(55), (80), (84), and (87) describe modulated nonlinear wave trains, particularly relevant for understanding Langmuir and Alfvén wave packets under periodic forcing. Hybrid and singular solutions, such as those in Eqs. (50), (51), and (95)–(98), reveal complex and mixed nonlinear behaviors, including singular structures.

The obtained solutions can be broadly categorized based on their structure and physical implications. Bellshaped solitons, such as those in Eqs. (19), (72), and (81), exhibit sech-type profiles and represent stable, localized wave packets that preserve their shape during propagation. Kink and anti-kink solitons, appearing in Eqs. (23), (76), and (88), possess tanh-type profiles that model domain-wall-like transitions between different plasma states. Periodic and quasi-periodic solutions—based on elliptic functions as in Eqs. (26)–(30), (40)–(55), (80), (84), and (87)—describe modulated nonlinear wave trains, particularly relevant for understanding Langmuir and Alfvén wave packets under periodic forcing. Hybrid and singular solutions, such as those in Eqs. (50), (51), and (95)–(98), reveal complex and mixed nonlinear behaviors, including singular structures.

The bell-shaped envelope in Eq. (19) is the canonical “Langmuir envelope soliton” profile reported in type-III solar burst source regions and in auroral/solar-wind measurements: localized wave packets accompanied by density cavities driven by the ponderomotive force (OTSI route). The exponential tail in Eq. (20) represents the evanescent envelope in the cavity periphery. Our straddled solutions (Eqs. (50)–(51)) capture the coexistence of localized spikes on modulated backgrounds typical of strong Langmuir turbulence (Zakharov-type collapse stages) and can model intermittent packet trains across the heliosphere. Also, Eqs. (50)–(51) offer analytic templates for modulated Alfvénic structures in both space and fusion plasmas.

Across all families, derivative nonlinearities $\left(c_{1},c_{2},c_{3}\right)$ primarily control shape asymmetry, chirp, and effective group velocity, whereas algebraic nonlinearities $\left(b_{1},b_{2}\right)$ ​ set the amplitude–width trade-off and saturation. Signs of (C,E) choose the family; magnitudes of $\left(c_{1},c_{2},c_{3},b_{1},b_{2}\right)$ ​ tune the profile within that family. These trends persist across the bell (Eq. (19)), kink (Eq. (20)), and elliptic (Eqs. (50)–51) solutions, demonstrating robustness of the solution set under parameter variations.

The generalized model’s capacity to reproduce established DNLS-type equations in limiting cases reinforces its physical significance. The diversity of soliton structures reflects the rich nonlinear dynamics inherent in plasma systems, including wave localization, modulational instability, and potential collapse. Furthermore, the explicit dependence of the solutions on model parameters a, c1, c2, c3, b1, b2, along with constraints provided in Eqs. (8), (18), (22), (25), (32), (57), (61), (64), (71), (75), (79), (83), (86), (90), and (94), enhances the model’s applicability across diverse plasma environments. This parameter tunability allows capturing effects such as self-steepening, higher-order nonlinearities, and amplitude–phase coupling. The complementary use of the modified sub-ODE and enhanced direct algebraic methods enabled comprehensive exploration of the solution space. The sub-ODE technique proved particularly effective in obtaining polynomial-type expressions, whereas the enhanced algebraic method enabled the derivation of more general solutions involving reciprocals of elliptic functions. The use of balancing principles ensured mathematical consistency, and the presence of special functions such as Weierstrass and Jacobi elliptic functions deepened the connection between nonlinear wave theory and classical function theory.

Although the analytical solutions presented offer valuable insight into the dynamics governed by the concatenated model, they are derived under specific ansatzes and parameter restrictions. Broader investigations such as general initial-boundary value problems, perturbations, and multi-soliton interactions remain open. Future research could include the derivation of conservation laws and Hamiltonian structures, extensions to fractional time evolution and nonlocal operators, and numerical analysis using techniques like the Adomian Decomposition Method (ADM), Improved ADM, and the Variational Iteration Method (VIM). Moreover, examining hybrid and singular solitons in experimental plasma setups may bridge the gap between theory and physical realization.

To further illustrate the dynamics of the localized envelope soliton derived in Eq. (72), Fig. 1 visualizes its temporal and spatial evolution. The soliton exhibits a robust bell-shaped profile governed by a hyperbolic secant function, indicating strong spatial localization and temporal stability. Panel (a) presents a 3D surface plot of the intensity $|q\left(x,t\right){|}^{2}$, demonstrating shape preservation during propagation. Panel (b) offers a color-coded density map that clearly tracks the solitons trajectory. Panel (c) shows profile snapshots at multiple time instants, confirming invariant amplitude and velocity-hallmarks of a traveling envelope soliton in nonlinear dispersive media.

Similarly, Fig. 2 illustrates the kink-type soliton governed by Eq. (76), which features a sharp transition between two asymptotic states. The 3D plot in Panel (a) shows a monotonically increasing wavefront. The colormap in Panel (b) captures the advancing kink front across time, while Panel (c) shows the evolution of the wave profile, maintaining steepness and velocity. This behavior is characteristic of topological or domain-wall solitons, often observed in nonlinear plasma systems and bistable media.

Many nonlinear plasma phenomena are traditionally modeled by separate DNLS-type equations, notably the Kaup–Newell (KN), Chen–Lee–Liu (CLL), and Gerdjikov–Ivanov (GI) forms. Our concatenated model provides a single parameterized framework in which these equations arise as embedded limits, allowing analysts to traverse regimes by tuning to $\left(c_{1},c_{2},c_{3},b_{1},b_{2}\right)$ rather than switching models. This produces a unified reduced ODE solved with two complementary techniques, yielding Weierstrass- and Jacobi-based solution families and a coherent taxonomy of plasma structures.

The model consolidates dynamics relevant to Langmuir waves, Alfvén waves, and cold-plasma settings, offering one tractable formulation for disparate regimes. The derivative couplings to $\left(c_{1},c_{2},c_{3}\right)$ encode convective self-steepening and mixed derivative nonlinearities that arise in magnetized plasmas due to velocity-dependent phase modulation and higher-order nonlinear dispersion. The saturation terms to $\left(b_{1},b_{2}\right)$ regulate strong-amplitude behavior, preventing unbounded growth and adjusting background equilibria. In practice, this parameterization enables controlled exploration of dispersive–nonlinear balances that shape envelope formation, steepening, and saturation in these media.

The derivative couplings $\left(c_{1},c_{2},c_{3}\right)$ encode convective self-steepening and mixed derivative nonlinearities that arise in magnetized plasmas due to velocity-dependent phase modulation and higher-order nonlinear dispersion. Solitary waves (sech-type) and Kink-type waves (tanh-type) envelopes correspond to localized density depressions and enhancements in Langmuir wave packets, while kink/anti-kink structures represent transition layers and current sheets. Periodic cn/dn/sn wavetrains capture modulated backgrounds observed in weak turbulence. The quintic term $\left(b_{2}\right)$ provides saturation that prevents unbounded growth in strong-amplitude regimes and adjusts the equilibrium background. We outline parameter windows consistent with DNLS reductions (weakly collisional electron-ion plasmas, moderate magnetization, narrowband envelopes) and suggest diagnostics (envelope speed, chirp, skewness) for laboratory and space applications.

For each solution set, admissibility is governed by the signs of (C,E), the positivity of $A_{0}$, and the coupling relations that connect (a,κ,ω,V) to $\left(c_{1},c_{2},c_{3},b_{1},b_{2}\right)$ via Eq. (8) and the coefficients $\Delta_{j}$. When C>0 and E<0, the reduced ODE supports solitary waves (sech-type) envelopes; when C<0 and E>0, Kink-type waves (tanh-type) and anti-kink branches emerge; Jacobi cn/dn/sn families arise for |m|<1 with the corresponding limits $m\to 1^{-}$ recovering solitary waves. Singular and hybrid/straddled forms belong to limiting or non-regular branches and should be interpreted cautiously depending on physical regularization.

Amplitude A and width W scale with the effective dispersive and nonlinear coefficients in the reduced ODE. Increasing the magnitude of the derivative nonlinearity (through $c_{1},c_{2},c_{3}$) sharpens profiles (reduced W) and can flip polarity under sign changes of C and E; increasing $b_{2}$ saturates the envelope and elevates the background level. The velocity V=−2aκ links dispersion a and carrier wavenumber κ and is therefore tunable independently of envelope amplitude through the coupling constraint.

We verify closed-form solutions by symbolic back-substitution into the reduced ODE and the original PDE, confirming vanishing residuals under the stated constraints. As a potential direction for future work, the model could be initialized with a closed-form envelope and evolved using a split-step Fourier method. By monitoring the $L^{2}$ norm and peak amplitude during propagation, one could quantitatively assess the stability and robustness of the scheme within the admissible parameter ranges.

While direct numerical or experimental validation is left for future work, we propose a clear testing pathway. In practice, spacecraft E-field measurements of Langmuir bursts or auroral solitons can be fitted to Eq. (19), while magnetotail reconnection layers can be compared with the tanh-type profile of Eq. (20). In fusion experiments, diagnostics such as Mirnov coils or reflectometry could be used to search for wavetrain signatures analogous to the elliptic solutions (Eqs. (50)–51).

## Limitations

The current paper first formulated a new form of the concatenation model from the three models that are from plasma physics. Therefore this model combines the effect of Alfvén waves, Langmuir waves and cold plasmas to address their cumulative effect. The model is formulated along the same lines as the concatenation model and the dispersive concatenation model from optics. The solutions to the solitons are obtained using two types of integration architectures that produced the necessary solutions via the intermediate solutions, which are the Weierstrass’ and Jacobi’s elliptic functions. Both the hybrid and single soliton solutions are recovered and used in the project. Thus, the proposed model holds significant promise for future developments in plasma physics. A number of the model’s features will be adopted in the future. These include retrieval of the conservation laws for the model, locating the quiescent solitons for nonlinear dispersion, integrating the model with fractional temporal evolution, numerical solutions to the model by implementing the Adomian decomposition method (ADM), improved ADM and the Laplace–ADM scheme, variational iteration approach and many others.

In summary, we introduce a concatenated derivative nonlinear Schrödinger (DNLS) model for plasma physics that, for the first time, unifies the Kaup–Newell, Chen–Lee–Liu, and Gerdjikov–Ivanov equations within a single parametric framework. By applying the enhanced direct algebraic method and the modified sub-ODE approach, we derive broad families of coherent structures—bell-shaped, kink-type, hybrid, and straddled solitons—expressed through Jacobi and Weierstrass elliptic functions. This tunable formulation connects the derivative couplings $\left(c_{1},c_{2},c_{3}\right)$ and higher-order amplitudinal terms $\left(b_{1},b_{2}\right)$ to physically distinct regimes (convective self-steepening, mixed derivative nonlinearities, quintic saturation), enabling controlled passage between embedded limits and a unified taxonomy of plasma waves relevant to Langmuir, Alfvén, and cold-plasma environments. These results clarify the model’s novelty, integrability under explicit constraints, and utility as a compact tool for analyzing localized wave dynamics across multiple plasma contexts.

## Ethics statements

This work does not involve human subjects or animal experiments. There are no ethical concerns related to this study.

## CRediT authorship contribution statement

**Elsayed M.E. Zayed:** Conceptualization, Supervision, Writing – Review & Editing. **Mona El–Shater:** Methodology, Software, Formal Analysis, Writing – Original Draft. **Ahmed H. Arnous:** Validation, Visualization, Data Curation, Writing – Review & Editing. **Anjan Biswas:** Conceptualization, Methodology, Writing – Review & Editing, Funding Acquisition, Supervision.

## Declaration of competing interest

The authors declare that they have no known competing financial interests or personal relationships that could have appeared to influence the work reported in this paper.

## Data Availability

No data was used for the research described in the article.
